# Di-μ-thio­cyanato-κ^2^ *N*:*S*;κ^2^ *S*:*N*-bis­[bis­(2-methyl-1*H*-benzimidazole-κ*N* ^3^)(thio­cyanato-κ*N*)cadmium(II)]

**DOI:** 10.1107/S1600536810042443

**Published:** 2010-10-23

**Authors:** Shayma A. Shaker, Hamid Khaledi, Hapipah Mohd Ali

**Affiliations:** a Department of Chemistry, University of Malaya, 50603 Kuala Lumpur, Malaysia

## Abstract

The title compound, [Cd_2_(NCS)_4_(C_8_H_8_N_2_)_4_], is a centrosymmetric dinuclear cadmium(II) complex in which each two metal ions are linked by a pair of thio­cyanate *N*:*S*-bridges. Two 2-methyl­benzimidazole N-atom donors and one terminal thio­cyanate N atom complete a highly distorted square-pyramidal geometry around the Cd atom. In the crystal structure, two N—H⋯S hydrogen-bonding inter­actions occur, resulting in a three-dimensional polymeric structure. The apical 2-methyl­benzimidazole ring and its symmetry-related counterpart are arranged in an anti­parallel manner with a centroid–centroid separation of 3.6050 (14) Å, indicative of a π–π inter­action.

## Related literature

For cadmium complexes having a [Cd_2_(μ_2_ *-*NCS)_2_(NCS)_2_] unit, see: Gou *et al.* (2008 ▶); Govor *et al.* (2008 ▶); Shi *et al.* (2004 ▶).
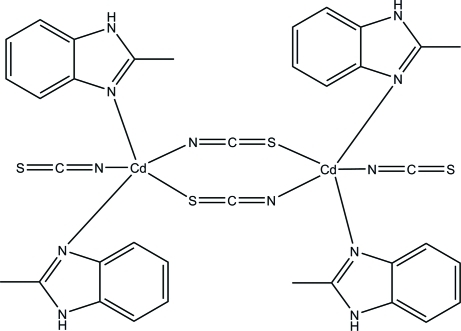


## Experimental

### 

#### Crystal data

[Cd_2_(NCS)_4_(C_8_H_8_N_2_)_4_]
*M* *_r_* = 985.78
Monoclinic, 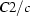

*a* = 18.1519 (11) Å
*b* = 10.2098 (6) Å
*c* = 21.7385 (13) Å
β = 97.864 (1)°
*V* = 3990.8 (4) Å^3^
*Z* = 4
Mo *K*α radiation
μ = 1.32 mm^−1^
*T* = 100 K
0.38 × 0.20 × 0.13 mm

#### Data collection

Bruker APEXII CCD diffractometer
Absorption correction: multi-scan (*SADABS*; Sheldrick, 1996 ▶) *T*_min_ = 0.634, *T*_max_ = 0.847
9361 measured reflections
3593 independent reflections
3213 reflections with *I* > 2σ(*I*)
*R*_int_ = 0.022

#### Refinement

*R*[*F* ^2^ > 2σ(*F* ^2^)] = 0.021
*wR*(*F* ^2^) = 0.047
*S* = 1.03
3593 reflections
253 parameters
3 restraints
H atoms treated by a mixture of independent and constrained refinement
Δρ_max_ = 0.35 e Å^−3^
Δρ_min_ = −0.31 e Å^−3^

### 

Data collection: *APEX2* (Bruker, 2007 ▶); cell refinement: *SAINT* (Bruker, 2007 ▶); data reduction: *SAINT*; program(s) used to solve structure: *SHELXS97* (Sheldrick, 2008 ▶); program(s) used to refine structure: *SHELXL97* (Sheldrick, 2008 ▶); molecular graphics: *XP* in *SHELXTL* (Sheldrick, 2008 ▶); software used to prepare material for publication: *SHELXL97* and *publCIF* (Westrip, 2010 ▶).

## Supplementary Material

Crystal structure: contains datablocks I, global. DOI: 10.1107/S1600536810042443/pk2278sup1.cif

Structure factors: contains datablocks I. DOI: 10.1107/S1600536810042443/pk2278Isup2.hkl

Additional supplementary materials:  crystallographic information; 3D view; checkCIF report

## Figures and Tables

**Table 1:** Hydrogen-bond geometry (Å, °)

*D*—H⋯*A*	*D*—H	H⋯*A*	*D*⋯*A*	*D*—H⋯*A*
N2—H2*N*⋯S1^i^	0.86 (2)	2.45 (2)	3.273 (2)	163 (2)
N4—H4*N*⋯S1^ii^	0.84 (2)	2.48 (2)	3.281 (2)	159 (2)

Symmetry codes: (i) 
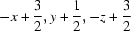
; (ii) 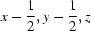.

**Fig. 1.**
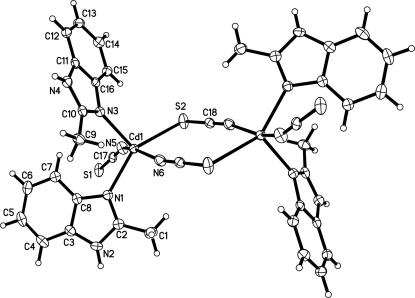
The molecular structure of the title compound showing displacement ellipsoids at the 50% probability level. Hydrogen atoms are drawn as spheres of arbitrary radius. The unlabelled atoms are generated by the symmetry operation (–x + 1, –y + 1, –z + 1).

**Fig. 2.**
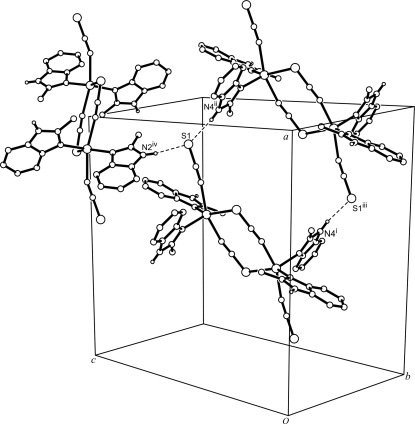
A view of the hydrogen bonding interactions (dashed lines). C-bound hydrogen atoms have been omitted for clarity. Symmetry codes: (i) -x + 1, -y + 1, -z + 1; (ii) x + 1/2, y + 1/2, z; (iii) -x + 3/2, -y + 3/2, -z + 1/2; (iv) -x+3/2, y - 1/2, - z+3/2.

## supplementary crystallographic information

### Comment

The title compound is a mixed-ligand cadmium(II) complex with thiocyanate and 2-methylbenzimidazole. The thiocyanate ions act as either bridging or terminal ligands. The pairs of the former, link each two metal ions into a centrosymmetric dinuclear complex. The resulting eight-membered Cd_2_(µ_2_*-*NCS)_2 _ring is almost planar (r.m.s = 0.015), unlike the chair conformation observed in most of the similar structures (Gou *et al.* 2008; Govor *et al.* 2008; Shi *et al.* 2004). The terminal thiocyanate ligand forms two types of hydrogen bond with the amine hydrogen atoms (Fig. 2), leading to an infinite three-dimensional network. Two 2-methylbenzimidazole ligands, one in the apical position and the other one equatorially positioned, complete the penta-coordinate cadmium(II) complex in a highly distorted square pyramidal geometry (τ = 0.26).  The apical 2-methylbenzimidazole ring and its symmetry related counterpart at (-x+1, -y, -z+1) are arranged in an antiparallel manner with separation of 3.6050 (14) Å, indicative of a π-π interaction.

### Experimental

An ethanolic solution (15 ml) of 2-methylbenzimidazole (1.32 g, 10 mmol) was added to an aqueous solution (20 ml) of CdCl_2_. H_2_O (0.5 g, 2.5 mmol) followed by addition of an aqueous solution (15 ml) of KSCN (0.49 g, 5 mmol). The mixture was heated in a water bath for 30 min with constant stirring. The resulting precipitate was filtered off and recrystallized from ethanol to give the colorless crystals of the title cadmium(II) complex.

### Refinement

The C-bound hydrogen atoms were placed in idealized locations (C—H = 0.95–0.98 Å) and refined as riding on their parent carbon atoms. The N-bound hydrogen atoms were located in a difference Fourier map and were refined with distance restraints of N—H 0.88 (2) Å. *U*(H) were set to either 1.2 *U*eq of the parent atom, or 1.5 *U*eq(C) for methyl H. An additional rigid-bond type restraint (DELU in SHELXL97) was placed on the displacement parameters of S1 and C17.

### Crystal data

**Table:**

[Cd_2_(NCS)_4_(C_8_H_8_N_2_)_4_]	*F*(000) = 1968
*M**_r_* = 985.78	*D*_x_ = 1.641 Mg m^−^^3^
Monoclinic, *C*2/*c*	Mo *K*α radiation, λ = 0.71073 Å
Hall symbol: -C 2yc	Cell parameters from 4666 reflections
*a* = 18.1519 (11) Å	θ = 2.3–30.1°
*b* = 10.2098 (6) Å	µ = 1.32 mm^−^^1^
*c* = 21.7385 (13) Å	*T* = 100 K
β = 97.864 (1)°	Block, colorless
*V* = 3990.8 (4) Å^3^	0.38 × 0.20 × 0.13 mm
*Z* = 4	

### Data collection

**Table:**

Bruker APEXII CCD diffractometer	3593 independent reflections
Radiation source: fine-focus sealed tube	3213 reflections with *I* > 2σ(*I*)
graphite	*R*_int_ = 0.022
φ and ω scans	θ_max_ = 25.2°, θ_min_ = 2.3°
Absorption correction: multi-scan (*SADABS*; Sheldrick, 1996)	*h* = −21→21
*T*_min_ = 0.634, *T*_max_ = 0.847	*k* = −12→8
9361 measured reflections	*l* = −26→26

### Refinement

**Table:**

Refinement on *F*^2^	Primary atom site location: structure-invariant direct methods
Least-squares matrix: full	Secondary atom site location: difference Fourier map
*R*[*F*^2^ > 2σ(*F*^2^)] = 0.021	Hydrogen site location: inferred from neighbouring sites
*wR*(*F*^2^) = 0.047	H atoms treated by a mixture of independent and constrained refinement
*S* = 1.03	*w* = 1/[σ^2^(*F*_o_^2^) + (0.0169*P*)^2^ + 6.0676*P*] where *P* = (*F*_o_^2^ + 2*F*_c_^2^)/3
3593 reflections	(Δ/σ)_max_ = 0.003
253 parameters	Δρ_max_ = 0.35 e Å^−^^3^
3 restraints	Δρ_min_ = −0.31 e Å^−^^3^

### Special details

**Table:**

Geometry. All e.s.d.'s (except the e.s.d. in the dihedral angle between two l.s. planes) are estimated using the full covariance matrix. The cell e.s.d.'s are taken into account individually in the estimation of e.s.d.'s in distances, angles and torsion angles; correlations between e.s.d.'s in cell parameters are only used when they are defined by crystal symmetry. An approximate (isotropic) treatment of cell e.s.d.'s is used for estimating e.s.d.'s involving l.s. planes.
Refinement. Refinement of *F*^2^ against ALL reflections. The weighted *R*-factor *wR* and goodness of fit *S* are based on *F*^2^, conventional *R*-factors *R* are based on *F*, with *F* set to zero for negative *F*^2^. The threshold expression of *F*^2^ > σ(*F*^2^) is used only for calculating *R*-factors(gt) *etc*. and is not relevant to the choice of reflections for refinement. *R*-factors based on *F*^2^ are statistically about twice as large as those based on *F*, and *R*- factors based on ALL data will be even larger.

### Fractional atomic coordinates and isotropic or equivalent isotropic displacement parameters (Å^2^)

**Table:**

	*x*	*y*	*z*	*U*_iso_*/*U*_eq_	
Cd1	0.611050 (9)	0.354819 (16)	0.590885 (7)	0.01561 (6)	
S1	0.88100 (3)	0.33657 (6)	0.67126 (3)	0.02608 (15)	
S2	0.63546 (3)	0.42113 (7)	0.47897 (3)	0.02741 (16)	
N1	0.62322 (10)	0.41831 (19)	0.69141 (9)	0.0181 (4)	
N2	0.63781 (11)	0.5391 (2)	0.77688 (9)	0.0211 (5)	
H2N	0.6364 (14)	0.609 (2)	0.7982 (11)	0.025*	
N3	0.57608 (10)	0.14637 (19)	0.58946 (8)	0.0166 (4)	
N4	0.50684 (11)	−0.0294 (2)	0.59628 (9)	0.0193 (4)	
H4N	0.4715 (12)	−0.075 (2)	0.6062 (11)	0.023*	
N5	0.73824 (11)	0.3290 (2)	0.60377 (10)	0.0258 (5)	
N6	0.49556 (11)	0.4528 (2)	0.57293 (9)	0.0227 (5)	
C1	0.57531 (15)	0.6482 (2)	0.68162 (12)	0.0279 (6)	
H1A	0.5852	0.6462	0.6384	0.042*	
H1B	0.5953	0.7293	0.7014	0.042*	
H1C	0.5215	0.6444	0.6826	0.042*	
C2	0.61150 (12)	0.5342 (2)	0.71553 (11)	0.0195 (5)	
C3	0.67023 (13)	0.4198 (2)	0.79411 (11)	0.0199 (5)	
C4	0.70777 (13)	0.3741 (3)	0.85017 (11)	0.0256 (6)	
H4	0.7157	0.4277	0.8862	0.031*	
C5	0.73278 (13)	0.2465 (3)	0.85032 (11)	0.0267 (6)	
H5	0.7590	0.2115	0.8875	0.032*	
C6	0.72085 (13)	0.1674 (3)	0.79777 (11)	0.0244 (6)	
H6	0.7376	0.0791	0.8004	0.029*	
C7	0.68519 (13)	0.2141 (2)	0.74170 (11)	0.0207 (5)	
H7	0.6782	0.1606	0.7056	0.025*	
C8	0.66004 (12)	0.3433 (2)	0.74051 (10)	0.0180 (5)	
C9	0.47424 (14)	0.1670 (2)	0.65538 (11)	0.0235 (6)	
H9A	0.5070	0.1980	0.6921	0.035*	
H9B	0.4369	0.1074	0.6683	0.035*	
H9C	0.4493	0.2419	0.6333	0.035*	
C10	0.51909 (12)	0.0964 (2)	0.61350 (10)	0.0180 (5)	
C11	0.55900 (12)	−0.0656 (2)	0.55862 (10)	0.0179 (5)	
C12	0.57193 (13)	−0.1818 (2)	0.52880 (11)	0.0215 (5)	
H12	0.5417	−0.2570	0.5316	0.026*	
C13	0.63096 (14)	−0.1828 (2)	0.49486 (11)	0.0224 (5)	
H13	0.6421	−0.2610	0.4743	0.027*	
C14	0.67477 (13)	−0.0712 (2)	0.49010 (11)	0.0221 (5)	
H14	0.7146	−0.0752	0.4660	0.027*	
C15	0.66144 (13)	0.0441 (2)	0.51961 (10)	0.0191 (5)	
H15	0.6914	0.1194	0.5164	0.023*	
C16	0.60252 (12)	0.0460 (2)	0.55436 (10)	0.0162 (5)	
C17	0.79736 (13)	0.3327 (2)	0.63127 (11)	0.0190 (5)	
C18	0.55777 (13)	0.4945 (2)	0.44892 (10)	0.0181 (5)	

### Atomic displacement parameters (Å^2^)

**Table:**

	*U*^11^	*U*^22^	*U*^33^	*U*^12^	*U*^13^	*U*^23^
Cd1	0.01419 (9)	0.01624 (10)	0.01592 (9)	−0.00017 (7)	0.00037 (6)	−0.00061 (7)
S1	0.0177 (3)	0.0296 (4)	0.0292 (3)	−0.0058 (3)	−0.0026 (3)	0.0120 (3)
S2	0.0179 (3)	0.0383 (4)	0.0273 (3)	0.0099 (3)	0.0080 (3)	0.0139 (3)
N1	0.0166 (10)	0.0187 (11)	0.0187 (10)	0.0004 (8)	0.0009 (8)	−0.0010 (9)
N2	0.0222 (11)	0.0219 (12)	0.0193 (11)	0.0006 (9)	0.0026 (9)	−0.0070 (9)
N3	0.0148 (9)	0.0179 (10)	0.0169 (9)	−0.0008 (8)	0.0016 (8)	0.0001 (8)
N4	0.0176 (10)	0.0214 (11)	0.0190 (10)	−0.0059 (8)	0.0024 (8)	0.0023 (9)
N5	0.0183 (11)	0.0351 (13)	0.0245 (11)	0.0003 (9)	0.0043 (9)	0.0033 (10)
N6	0.0210 (11)	0.0283 (12)	0.0188 (10)	0.0040 (9)	0.0031 (9)	−0.0008 (9)
C1	0.0305 (14)	0.0229 (14)	0.0288 (13)	0.0028 (11)	−0.0010 (12)	−0.0035 (12)
C2	0.0150 (11)	0.0213 (13)	0.0223 (12)	−0.0009 (10)	0.0027 (10)	−0.0012 (11)
C3	0.0160 (12)	0.0262 (14)	0.0181 (12)	−0.0029 (10)	0.0043 (10)	−0.0003 (11)
C4	0.0209 (13)	0.0399 (17)	0.0162 (12)	−0.0057 (11)	0.0033 (10)	−0.0021 (11)
C5	0.0186 (13)	0.0414 (17)	0.0202 (13)	0.0015 (11)	0.0023 (10)	0.0108 (12)
C6	0.0185 (12)	0.0260 (14)	0.0298 (14)	0.0029 (10)	0.0071 (11)	0.0095 (11)
C7	0.0177 (12)	0.0222 (14)	0.0222 (12)	0.0005 (10)	0.0027 (10)	0.0000 (11)
C8	0.0138 (11)	0.0215 (13)	0.0185 (12)	−0.0012 (10)	0.0018 (9)	0.0018 (10)
C9	0.0232 (13)	0.0284 (15)	0.0197 (12)	−0.0038 (11)	0.0061 (10)	−0.0028 (11)
C10	0.0164 (12)	0.0220 (13)	0.0148 (11)	−0.0014 (10)	−0.0002 (10)	0.0035 (10)
C11	0.0178 (12)	0.0211 (13)	0.0139 (11)	−0.0015 (10)	−0.0016 (9)	0.0039 (10)
C12	0.0248 (13)	0.0182 (13)	0.0198 (12)	−0.0046 (10)	−0.0025 (10)	0.0013 (10)
C13	0.0256 (13)	0.0197 (13)	0.0206 (12)	0.0024 (10)	−0.0010 (11)	−0.0029 (10)
C14	0.0194 (12)	0.0262 (14)	0.0208 (12)	0.0011 (10)	0.0032 (10)	−0.0010 (11)
C15	0.0174 (12)	0.0184 (13)	0.0210 (12)	−0.0023 (10)	0.0002 (10)	0.0004 (10)
C16	0.0156 (11)	0.0176 (12)	0.0141 (11)	0.0004 (9)	−0.0023 (9)	0.0016 (10)
C17	0.0201 (12)	0.0184 (13)	0.0193 (12)	−0.0006 (10)	0.0061 (10)	0.0050 (10)
C18	0.0177 (12)	0.0207 (13)	0.0164 (12)	−0.0015 (10)	0.0048 (10)	0.0004 (10)

### Geometric parameters (Å, °)

**Table:**

Cd1—N3	2.2199 (19)	C3—C8	1.394 (3)
Cd1—N1	2.2611 (19)	C4—C5	1.379 (4)
Cd1—N5	2.302 (2)	C4—H4	0.9500
Cd1—N6	2.307 (2)	C5—C6	1.392 (4)
Cd1—S2	2.6207 (7)	C5—H5	0.9500
S1—C17	1.644 (2)	C6—C7	1.385 (3)
S2—C18	1.651 (2)	C6—H6	0.9500
N1—C2	1.323 (3)	C7—C8	1.394 (3)
N1—C8	1.406 (3)	C7—H7	0.9500
N2—C2	1.355 (3)	C9—C10	1.488 (3)
N2—C3	1.382 (3)	C9—H9A	0.9800
N2—H2N	0.856 (17)	C9—H9B	0.9800
N3—C10	1.323 (3)	C9—H9C	0.9800
N3—C16	1.401 (3)	C11—C12	1.388 (3)
N4—C10	1.347 (3)	C11—C16	1.396 (3)
N4—C11	1.384 (3)	C12—C13	1.382 (3)
N4—H4N	0.844 (16)	C12—H12	0.9500
N5—C17	1.155 (3)	C13—C14	1.401 (3)
N6—C18^i^	1.152 (3)	C13—H13	0.9500
C1—C2	1.482 (3)	C14—C15	1.378 (3)
C1—H1A	0.9800	C14—H14	0.9500
C1—H1B	0.9800	C15—C16	1.391 (3)
C1—H1C	0.9800	C15—H15	0.9500
C3—C4	1.393 (3)	C18—N6^i^	1.152 (3)
			
N3—Cd1—N1	106.16 (7)	C4—C5—H5	118.9
N3—Cd1—N5	99.90 (7)	C6—C5—H5	118.9
N1—Cd1—N5	87.22 (7)	C7—C6—C5	121.6 (2)
N3—Cd1—N6	99.32 (7)	C7—C6—H6	119.2
N1—Cd1—N6	90.32 (7)	C5—C6—H6	119.2
N5—Cd1—N6	160.55 (7)	C6—C7—C8	117.1 (2)
N3—Cd1—S2	108.61 (5)	C6—C7—H7	121.5
N1—Cd1—S2	144.74 (5)	C8—C7—H7	121.5
N5—Cd1—S2	81.28 (5)	C3—C8—C7	120.6 (2)
N6—Cd1—S2	89.77 (5)	C3—C8—N1	108.9 (2)
C18—S2—Cd1	103.94 (8)	C7—C8—N1	130.5 (2)
C2—N1—C8	105.77 (19)	C10—C9—H9A	109.5
C2—N1—Cd1	129.77 (16)	C10—C9—H9B	109.5
C8—N1—Cd1	123.42 (15)	H9A—C9—H9B	109.5
C2—N2—C3	108.3 (2)	C10—C9—H9C	109.5
C2—N2—H2N	122.1 (18)	H9A—C9—H9C	109.5
C3—N2—H2N	129.4 (18)	H9B—C9—H9C	109.5
C10—N3—C16	106.11 (19)	N3—C10—N4	111.5 (2)
C10—N3—Cd1	126.99 (16)	N3—C10—C9	125.3 (2)
C16—N3—Cd1	126.17 (15)	N4—C10—C9	123.2 (2)
C10—N4—C11	108.57 (19)	N4—C11—C12	132.7 (2)
C10—N4—H4N	123.9 (18)	N4—C11—C16	105.1 (2)
C11—N4—H4N	127.4 (18)	C12—C11—C16	122.2 (2)
C17—N5—Cd1	154.70 (19)	C13—C12—C11	116.7 (2)
C18^i^—N6—Cd1	164.86 (18)	C13—C12—H12	121.7
C2—C1—H1A	109.5	C11—C12—H12	121.7
C2—C1—H1B	109.5	C12—C13—C14	121.5 (2)
H1A—C1—H1B	109.5	C12—C13—H13	119.2
C2—C1—H1C	109.5	C14—C13—H13	119.2
H1A—C1—H1C	109.5	C15—C14—C13	121.5 (2)
H1B—C1—H1C	109.5	C15—C14—H14	119.2
N1—C2—N2	111.7 (2)	C13—C14—H14	119.2
N1—C2—C1	126.1 (2)	C14—C15—C16	117.5 (2)
N2—C2—C1	122.2 (2)	C14—C15—H15	121.2
N2—C3—C4	132.2 (2)	C16—C15—H15	121.2
N2—C3—C8	105.3 (2)	C15—C16—C11	120.5 (2)
C4—C3—C8	122.4 (2)	C15—C16—N3	130.7 (2)
C5—C4—C3	116.1 (2)	C11—C16—N3	108.8 (2)
C5—C4—H4	121.9	N5—C17—S1	179.1 (2)
C3—C4—H4	121.9	N6^i^—C18—S2	178.5 (2)
C4—C5—C6	122.2 (2)		

Symmetry codes: (i) −*x*+1, −*y*+1, −*z*+1.

### Hydrogen-bond geometry (Å, °)

**Table:**

*D*—H···*A*	*D*—H	H···*A*	*D*···*A*	*D*—H···*A*
N2—H2N···S1^ii^	0.86 (2)	2.44 (2)	3.273 (2)	163 (2)
N4—H4N···S1^iii^	0.84 (2)	2.48 (2)	3.281 (2)	159 (2)

Symmetry codes: (ii) −*x*+3/2, *y*+1/2, −*z*+3/2; (iii) *x*−1/2, *y*−1/2, *z*.

## References

[bb1] Bruker (2007). *APEX2* and *SAINT* Bruker AXS Inc., Madison, Wisconsin, USA.

[bb2] Gou, L., Wu, Q. R., Hu, H. M., Qin, T., Xue, G. L., Yang, M. L. & Tang, Z. X. (2008). *Polyhedron*, **27**, 1517–1526.

[bb3] Govor, E. V., Lysenko, A. B., Domasevitch, K. V., Rusanov, E. B. & Chernega, A. N. (2008). *Acta Cryst.* C**64**, m117–m120. 10.1107/S0108270108001479 18322321

[bb4] Sheldrick, G. M. (1996). *SADABS* University of Göttingen, Germany.

[bb5] Sheldrick, G. M. (2008). *Acta Cryst.* A**64**, 112–122. 10.1107/S0108767307043930 18156677

[bb6] Shi, Q., Xu, L. J., Ji, J. X., Li, Y. M., Wang, R. H., Zhou, Z. Y., Cao, R., Hong, M. C. & Chan, A. S. C. (2004). *Inorg. Chem. Commun.* **7**, 1254–1257.

[bb7] Westrip, S. P. (2010). *J. Appl. Cryst.* **43**, 920–925.

